# Patient-Specific 3D-Printed Models and Mixed-Reality Head-Mounted Display Visualization for Aneurysm Clip Ligation Planning: A Methodological Workflow Study

**DOI:** 10.3390/brainsci16090932

**Published:** 2026-08-31

**Authors:** Joshua Haegler, Javier Anon, Gwendoline Canzanella, Stefanie Bauer, Debora Roethlisberger, Gerrit A. Schubert, Hans-Jakob Steiger, Luca Remonda, Serge Marbacher, Lukas Andereggen

**Affiliations:** 1 Department of Neurosurgery, Kantonsspital Aarau, 5001 Aarau, Switzerland; gwendoline.canzanella-boillat@szb-chb.ch (G.C.); stefanie.bauer@ksa.ch (S.B.); debora.roethlisberger@ksa.ch (D.R.); gerrit.schubert@ksa.ch (G.A.S.); hsteiger@uni-duesseldorf.de (H.-J.S.); neurochirurgie.marbacher@hin.ch (S.M.); 2 Department of Neurosurgery, TUM Medical School, Technical University of Munich, 81675 Munich, Germany; 3 Department of Radiology, Division of Neuroradiology, Kantonsspital Aarau, 5001 Aarau, Switzerland; javier.anon@ksa.ch (J.A.); luca.remonda@bluewin.ch (L.R.); 4 Department of Neurosurgery, RWTH Aachen University, 52062 Aachen, Germany; 5 Department of Neurosurgery, Heinrich-Heine-University, 40225 Düsseldorf, Germany; 6 Faculty of Medicine, University of Bern, 3012 Bern, Switzerland

**Keywords:** mixed reality, head-mounted display, 3D printing, cerebral aneurysm, aneurysm clipping, surgical planning

## Abstract

**Highlights:**

**What are the main findings?**

- We established a standardized patient-specific workflow for combined mixed-reality head-mounted display visualization and 3D-printed model generation in aneurysm clip ligation planning.
- The two modalities showed distinct workflow profiles: MR-HMD visualization enabled rapid three-dimensional review, whereas 3D-printed models provided a tangible planning and education tool but required longer production time.

**What are the implications of the main findings?**

- MR-HMD visualization and 3D-printed models should be considered complementary rather than competing tools in patient-specific neurovascular surgical planning.
- Modality selection should be guided by the clinical task and available preparation time, with MR-HMD suited for flexible short-term visualization and 3D printing suited for physical clip planning and patient education.

**Abstract:**

Background: Mixed-reality head-mounted display (MR-HMD) visualization and 3D-printed models may improve three-dimensional visualization in neurosurgical planning. Detailed, reproducible descriptions of the workflows, technical requirements, and practical limitations of these techniques in aneurysm clip ligation planning remain limited. Methods: Between February 2020 and June 2021, patient-specific MR-HMD visualizations and 3D-printed models were generated for 29 patients undergoing elective surgical clip ligation of intracranial aneurysms. CT and 3D rotational angiography were fused to create patient-specific holograms for MR-HMD visualization and 3D-printed models. All cases underwent standardized modality-specific processing. Preparation and manufacturing times as well as recorded workflow-related usability observations, technical advantages, and limitations were assessed. Results: The mean total preparation time (mean ± standard deviation, SD) was 40.6 ± 10.3 min for MR-HMD visualization and 108.8 ± 17.8 min for 3D-printed models, excluding printing, washing, and curing time. The average printing time for 3D-printed models was 894.9 ± 41.6 min. MR-HMD setup in the operating room averaged 7.0 ± 2.8 min. Identified limitations included manual hologram registration and repositioning after table or patient position changes, as well as the inability to track instruments or interact with the hologram. Conclusions: Both modalities could be integrated into a standardized patient-specific workflow and serve complementary functions. MR-HMD visualization supported flexible short-term visualization, whereas 3D-printed models provided a physical model for clip planning and patient education at the cost of longer preparation and production. These findings support a combined workflow in which modality selection is guided by clinical tasks and time constraints.

## 1. Introduction

Mixed reality (MR) describes the blending of real and virtual environments along the reality-virtuality continuum [1]. Over the past decade, MR-HMD visualization and 3D-printed models have increasingly been incorporated into neurosurgical applications, particularly for the visualization of neurovascular and neuro-oncological pathologies, surgical training, and preoperative simulation [2,3,4,5,6]. Several studies have demonstrated the feasibility of MR-HMD-based visualization for intraoperative visualization and navigation support [2,7,8,9,10,11,12], while 3D-printed anatomical models have been used for simulation, education, and patient-specific surgical planning [3,4,5,13,14,15].

Despite this growing adoption, detailed methodological reports on how to generate and implement patient-specific MR-HMD visualization and 3D-printed models for neurosurgical planning remain scarce. Moreover, only limited data are available on their combined or comparative use as visualization aids in the planning of intracranial aneurysm surgery [2,7,8,16]. To address this gap, the present study aims to establish and critically evaluate a reproducible and transferable methodological workflow for generating 3D-printed models and MR-HMD visualizations based on patient-specific imaging, and to assess their feasibility for integration into routine clinical practice. The methodological workflow was designed as a standardized framework comprising three predefined steps: first, acquisition and fusion of routinely obtained thin-slice CT and 3D rotational angiography datasets; second, modality-specific processing for MR-HMD visualization and 3D-printed models; and third, clinical integration into surgical planning, patient education, and non-sterile preoperative operating room setup. This structure was designed to provide a standardized, reproducible, and transferable framework for centers with comparable neurovascular imaging, MR-HMD visualization, and 3D-printing infrastructure.

## 2. Materials and Methods

### 2.1. Study Enrolment, Workup, and Ethics

We prospectively included 29 patients who underwent elective surgical clip ligation of an intracranial aneurysm between February 2020 and June 2021 at the Department of Neurosurgery, Kantonsspital Aarau. All patients were ≥18 years of age and provided written informed consent for participation and secondary use of anonymized data. The Human Research Ethics Committee of Nordwestschweiz approved the project on 23 September 2019 (EKNZ n°2019-01685).

### 2.2. Workflow Assessment

For each patient, preparation and manufacturing times as well as setup times for both techniques were recorded by stopwatch. Actual printing times were measured and reported by the printer software. Interruptions or parallel tasks were not documented separately; the reported times therefore represent elapsed time per step. MR-HMD setup time refers to the time required by the operator to launch the headset, load the hologram and manually align it with the patient’s head. Patient-specific MR-HMD visualizations and 3D-printed models were generated for 29 patients undergoing elective surgical clip ligation of intracranial aneurysms. MR-HMD visualization was prepared for all 29 patients but used preoperatively in 27 patients, because in 2 cases surgery was rescheduled and no member of the study team was available on the day of surgery to perform the MR-HMD setup. These two patients were therefore excluded from the MR-HMD setup-time analysis but not from the MR-HMD preparation time analysis and the 3D-printing analysis. Workflow-related technical advantages and limitations of MR-HMD visualization and 3D-printed models were assessed using structured evaluation forms completed per case (Supplementary Materials). For MR-HMD visualization, the form was completed by the neurosurgical resident assigned to the respective surgery (5 residents in total, each evaluating several cases). The evaluation forms were filled out directly after launching the MR-HMD and before surgery started. Evaluation forms were returned for all cases (response rate 100%). MR-HMD setup and conventional navigation setup were recorded on the same per-case form (Supplementary Materials) by the same neurosurgical resident. On the day of surgery, the resident entered the operating room during the anesthesiological preparation phase (patient positioning and placement of vascular access), launched the MR-HMD, loaded the hologram and aligned it with the patient’s head; the time from launching the device until the adapted hologram was displayed was recorded. Only thereafter did the routine neurosurgical preoperative preparation begin, including the setup of the conventional neuronavigation system (Brainlab Cranial Navigation, Brainlab AG, Munich, Germany), which was performed by the same resident according to clinical routine; the time from launching the navigation system until completion of patient registration was recorded in the same manner. Conventional navigation setup times were available for 27 patients. For the 3D-printed models, two neurosurgical residents and two attending neurosurgeons involved in neurovascular surgery evaluated the models of all 29 patients with one evaluation form per case for each. For the present methodological workflow study, only recorded setup times and open-text observations relating to feasibility, usability, and technical limitations of MR-HMD visualization and 3D-printed models were considered. Quantitative comparative ratings of different imaging and visualization modalities contained in the evaluation form (Supplementary Materials) were outside the scope of the present workflow analysis and were not analyzed or reported.

### 2.3. Workflow Standardization and Reproducibility

To enhance reproducibility, all cases were processed using Brainlab Cranial Planning (Elements Image Fusion Angio, Version 1.0 and Elements Image Fusion, Version 4.0, Brainlab AG) and Brainlab Mixed Reality Viewer software (Elements Viewer 5.0, Brainlab AG) in the versions available at study initiation in February 2020, in combination with Magic Leap One mixed-reality head-mounted display hardware (Magic Leap, Inc., Plantation, FL, USA) throughout the study period; 3D-printing preprocessing was performed using Formlabs PreForm version 3.3.3 initially, followed by software updates to versions 3.4.0, 3.4.1, and 3.10.1 during the study period, with all models manufactured using Formlabs Form 3 printers. The same sequence of image fusion, artifact removal, modality-specific processing, and clinical integration was applied to each patient. Operator assignment was fixed throughout the study. All digital preparation steps for the 3D-printed models (image registration, segmentation, delineation of perforators, STL generation, mesh editing, and print preparation) were performed by a single operator (J.A.). Printing, postprocessing, and painting of the models were performed by J.A. and J.H. Preprocessing for MR-HMD in Brainlab Cranial Planning software (Elements Image Fusion Angio, Version 1.0 and Elements Image Fusion, Version 4.0, Brainlab AG) was performed by J.H. Consequently, the preprocessing times reported in Table 1 reflect a single operator per modality (J.A. for the 3D-printed models, J.H. for MR-HMD visualization), whereas the postprocessing times for the 3D-printed models reflect the combined work of two operators. J.H. had no prior experience with 3D printing before this study.

### 2.4. Image Acquisition and Data Processing

All 3D-printed models and MR-HMD visualizations were generated from 3D rotational angiography (3D-RA) performed under local anesthesia and from thin-slice CT scans (1 mm), which were part of the routine preoperative evaluation. For MR-HMD visualization, imaging datasets were fused using Brainlab Cranial Planning software (Brainlab AG, Munich, Germany) to maximize resolution and visualize small perforating vessels.

#### 2.4.1. 3D-Printed Models

For each patient, a patient-specific STL file was generated from the 3D-RA aneurysm and CT skull DICOM data (3D Slicer, version 4.10.2, followed by updates during the study period: The Slicer Community; www.slicer.org, accessed on 3 February 2020) after spatial registration, segmentation, and manual definition of perianeurysmal perforators. Next, the aneurysm and skull base were sculpted, shaped, and hollowed using Meshmixer version 3.5 (Autodesk, Inc., San Rafael, CA, USA) and a plug-in adapter for the two components was implemented. Print preparation was performed in PreForm (Formlabs Inc., Somerville, MA, USA) and comprised printer setup, resin selection, printability verification, addition of supporting structures, and transfer of the STL file to a Form 3 printer. Manufacturing comprised printing at a layer height of 0.1 mm, washing in isopropyl alcohol for 20 min, and curing under heat and 405 nm light for 60 min (Form Wash and Form Cure, Formlabs Inc., Somerville, MA, USA). Postprocessing comprised manual removal of the supporting structures, painting, and assembly of the models.

**Figure 1:**
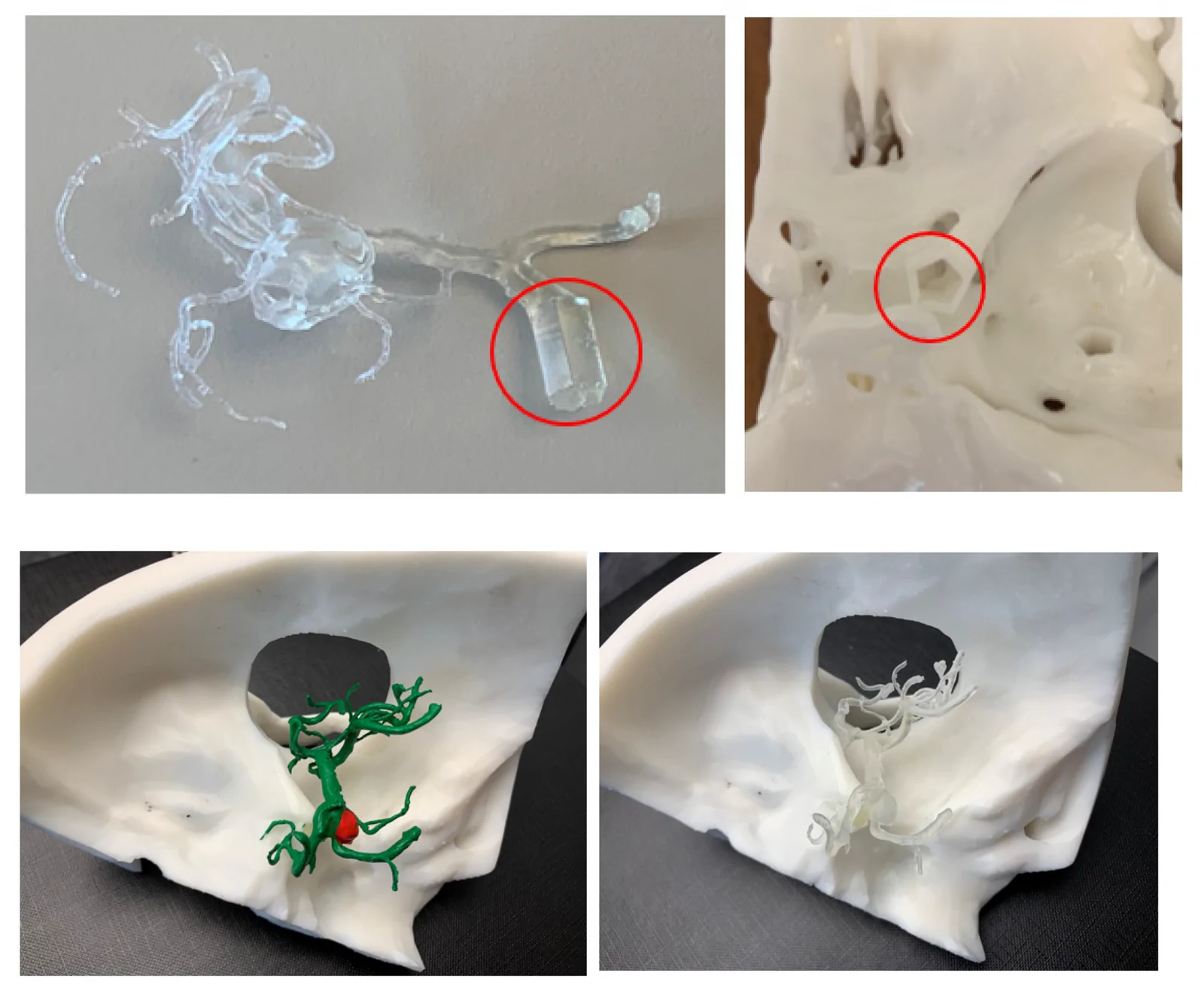
Translucent aneurysm model with hexagonal adapter (**upper left**, **red circle**) and matching counterpart in the skull model (**upper right**, **red circle**); assembled 3D-printed model with painted (**bottom left**) and unpainted (**bottom right**) aneurysm models.

For each patient, two aneurysm models were produced: one remained unpainted for surgical clip planning, and one was painted (aneurysms in red, arteries in green) for patient education (Figure 1).

Cost assessment was limited to 3D-printing materials and consumables using purchase prices during the study period. Recurring material and wear-part costs per patient-specific model set were analyzed, excluding one-time acquisition costs for printers, electricity costs, personnel costs, or software licensing fees; as production of both modalities was performed by multiple medical doctors in their free time, salaries would not be comparable internationally. Moreover, the software was either free of charge or already in use in our daily clinical routine, meaning no additional licensing fees were incurred. Reported costs were converted from Swiss francs to US dollars using a mean 2020–2021 exchange rate of 1 CHF = 1.08 USD.

#### 2.4.2. MR-HMD Visualization

Patient-specific holograms for MR-HMD visualization were generated from the fused CT and 3D-RA datasets using Brainlab Cranial Planning software. Preoperative processing included cropping of relevant anatomy and the delineation and removal of artifacts.

**Figure 2:**
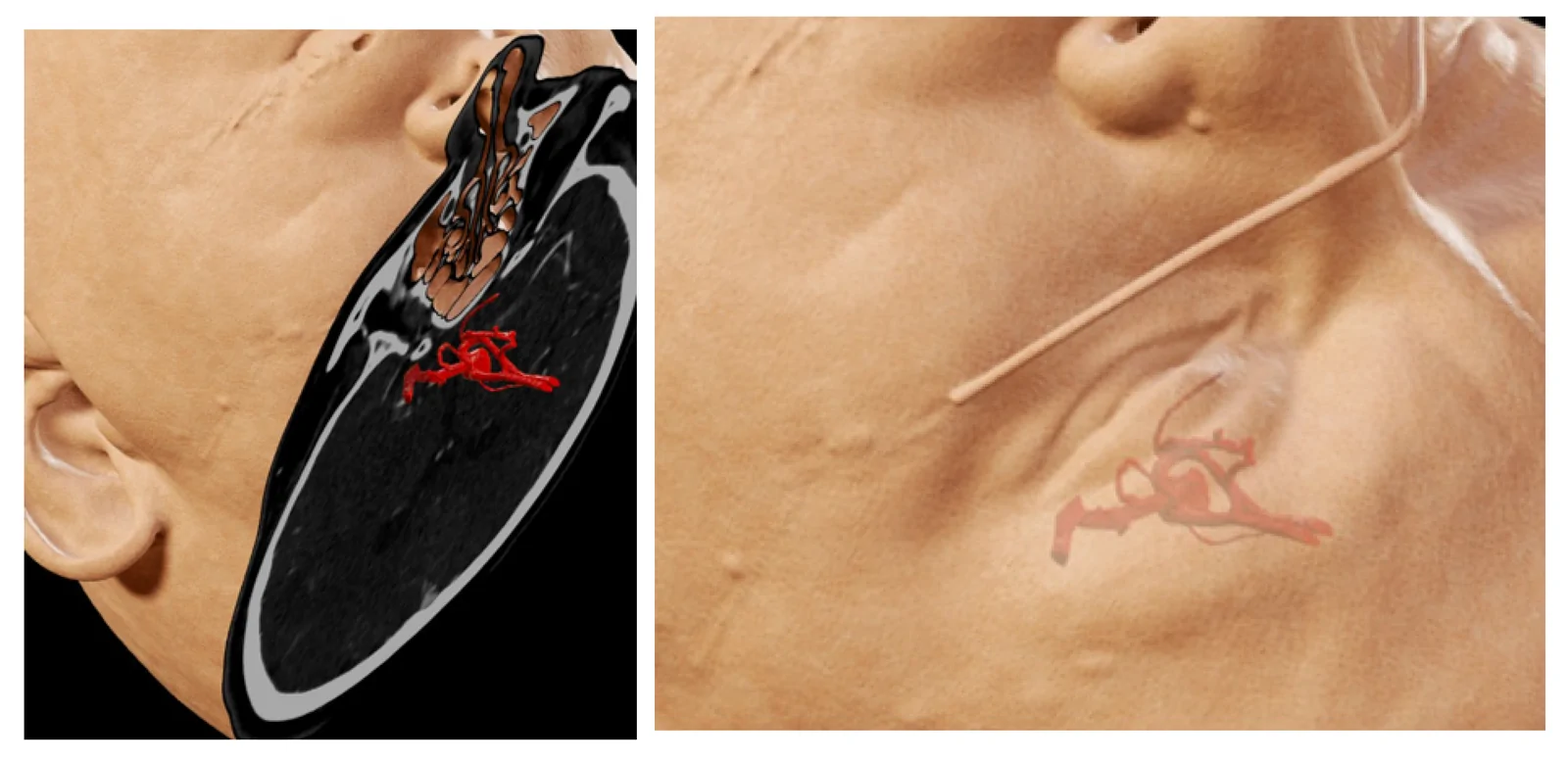
Patient-specific hologram generated in Brainlab Cranial Planning for MR-HMD visualization.

In the operating room, the MR-HMD (Magic Leap One; Magic Leap, Inc., Plantation, FL, USA) was launched using Brainlab Mixed Reality Viewer software (Brainlab AG, Munich, Germany), and the prepared patient-specific hologram was loaded by scanning a QR code generated by the planning software (Figure 2).

MR-HMD setup and manual alignment of the hologram with the patient’s head to assess spatial accuracy and correspondence with patient-specific anatomy were carried out via the headset concurrently with routine non-sterile operating room preparations (patient transfer, positioning, and anesthesia setup), in a time window that exists in every case (the anesthesiological preparation phase), and they were completed before the routine neurosurgical preoperative preparation started; the sterile phase of surgery was not affected, as the MR-HMD was not used after draping. This approach was chosen to minimize interference with the established workflow. The total duration of preoperative preparation with and without MR-HMD use was, however, not measured, and a minor delay cannot be formally excluded.

**Figure 3:**
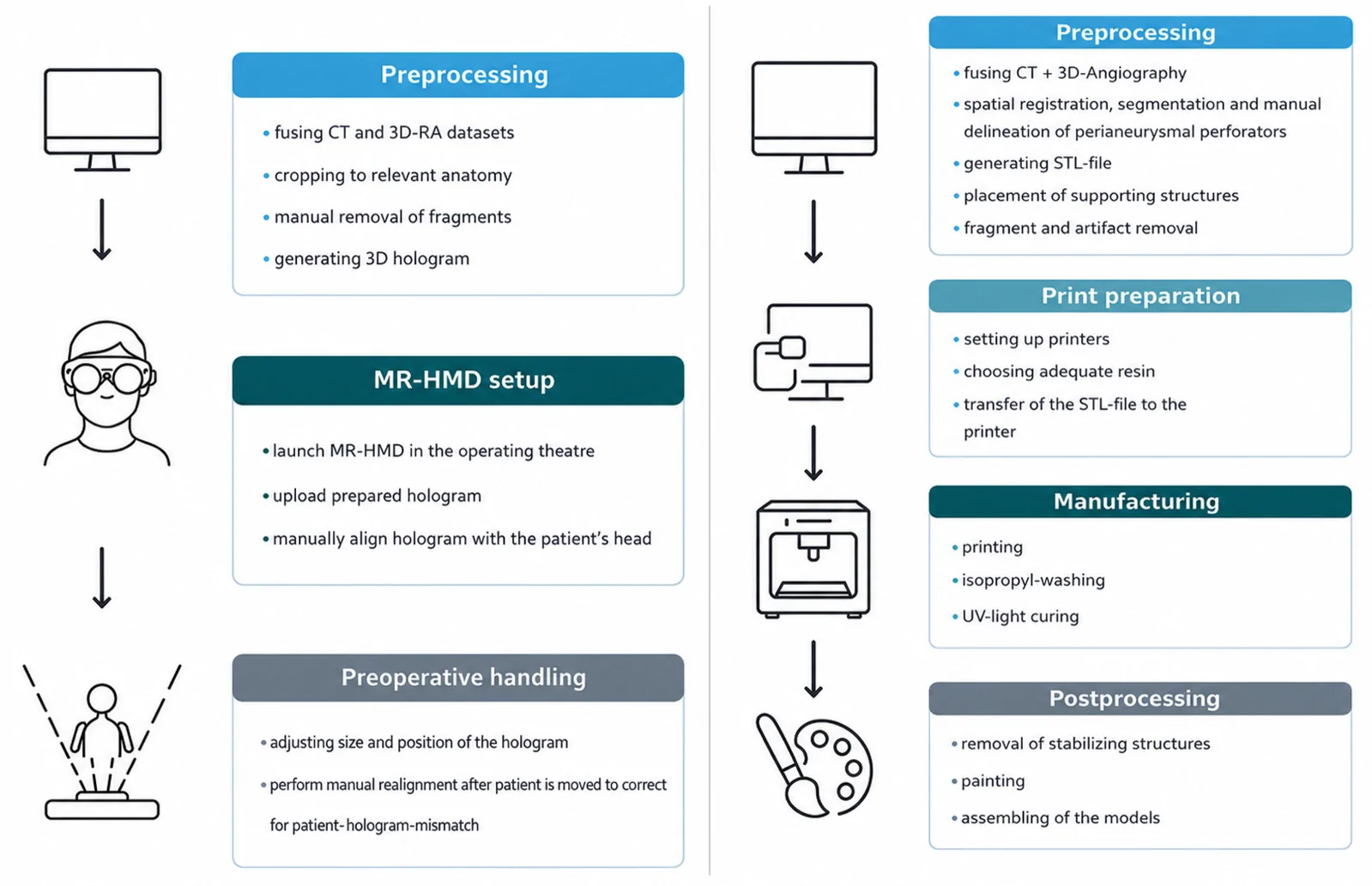
Flowchart for MR-HMD visualization (**left**) and 3D-printed model generation (**right**). Stage names correspond to those used in Section 2.4.1 and Table 1. Mesh editing and adapter design (Meshmixer) are part of preprocessing; placement of supporting structures was performed in PreForm as part of print preparation.

The complete workflows for MR-HMD visualization and 3D-printed model generation are summarized in Figure 3.

### 2.5. Statistical Analysis

Recorded time variables were analyzed descriptively and are reported as mean ± standard deviation (SD) and range (minimum–maximum). Open-text observations were reviewed descriptively to identify recurring workflow-related advantages and technical limitations of MR-HMD visualization and 3D-printed models. No inferential statistical testing was performed, as the present study was designed as a methodological workflow and feasibility assessment rather than a comparative efficacy study; the sample size and the non-randomized chronological case series design do not support hypothesis testing. Time trends across the case series were therefore examined descriptively. MR-HMD preprocessing time showed no apparent change across the case series, whereas postprocessing time for the 3D-printed models tended to be shorter in the later cases. Because postprocessing of the 3D-printed models was shared between two operators without per-case attribution of the individual contributions, no operator-specific analysis was possible, and the reported postprocessing times reflect the combined work of both operators.

## 3. Results

Total preparation time was shorter for MR-HMD visualization than for 3D-printed models. The mean total preparation time for MR-HMD visualization, combining preprocessing and setting up in the OR, was 40.6 ± 10.3 min (Table 1). Setup time in the OR for MR-HMD was 7.0 ± 2.8 min, comparable to 6.7 ± 2.1 min for conventional navigation setup measured in the same cohort (Brainlab Cranial Navigation, Brainlab AG, Munich, Germany). Total preparation time for 3D-printed models was 108.8 ± 17.8 min, excluding printing, washing, and curing time. The average printing time for the 3D-printed models was 14 h 55 min ± 41.6 min.

Preprocessing required similar times for both methods (41.0 ± 9.7 min for 3D-printed models vs. 33.5 ± 10.8 min for MR-HMD). Postprocessing for the 3D-printed models took 67.8 ± 12.4 min.

Reported advantages and disadvantages of both modalities are summarized in Table 2.

Limitations of the MR-HMD visualization included manual alignment of the hologram with the patient’s head prior to draping and repeated realignment after any movement of the operating table, despite rigid head fixation. Additional limitations in the hypothetical scenario of sterile intraoperative use of such MR-HMD holograms would include the lack of instrument tracking or interaction, limited visualization of small perforating vessels, and dependence of hologram resolution and performance on a stable internet connection.

For 3D-printed models, the main disadvantages were prolonged printing and restricted print size. Due to the limited build volume of the printer, only partial skull models were manufactured at life-size scale. Scaling down to enable complete skull printing was deliberately avoided in order to preserve original proportions, which were considered essential for clip planning.

Postprocessing time for the 3D-printed models tended to be shorter in the later cases of the series, which is compatible with increasing routine in the manual postprocessing steps. Given the descriptive design of this study and the shared operator assignment for these steps, this observation was not quantified further.

### Consumables and Wear-Part Costs

At the time of the study, material costs for 3D printing were as follows: a reusable resin tank cost USD 180. White rigid resin for skull models was USD 180 per liter, sufficient for approximately 3–4 skull prints, while elastic translucent resin for aneurysm models was USD 260 per liter, yielding 16–20 models. Additional consumables included isopropyl alcohol for postprocessing, paint for highlighting aneurysms, and minor replacement parts.

The building platforms required for the printing process showed wear after approximately 15 skull prints; each platform cost USD 130, and two replacements were necessary during the study period. Wear was greater for larger skull models compared to smaller aneurysm models.

Based on these values, the estimated average material and wear-part cost per patient for producing one skull model and two aneurysm models, one of which was painted, was approximately USD 130–150.

## 4. Discussion

This study establishes a standardized methodological workflow for integrating MR-HMD visualization and 3D-printed models into patient-specific aneurysm surgery planning. The two modalities showed distinct time profiles. Preparation of 3D-printed models was approximately 2.7-fold more time-consuming than preparation of MR-HMD visualization, and printing added substantial additional production time (Table 1). These data support the interpretation that MR-HMD visualization is better suited for flexible short-term visualization, whereas 3D-printed models provide a physical planning and education tool at the cost of longer elapsed production time. Accordingly, both modalities should be regarded as complementary rather than competing components of a patient-specific planning workflow.

Clinically, both modalities were integrated into preoperative planning, resident education, and patient education, but they contributed different modality-specific advantages and disadvantages, as reflected in the structured evaluation forms.

For 3D-printed models, reported advantages included improved visualization of small perforating vessels as well as tactile interaction for clip anticipation. Their main limitations were printing time, restricted print size, and the inability to visualize anatomical structures beyond bone and vessels. For MR-HMD visualization, reported advantages included add-on use on the patient during the non-sterile preoperative phase and adjustable visualization of additional patient-specific anatomical structures beyond the bone and vascular structures represented in the 3D-printed models, using zooming, rotation, and addition or removal of structures. Reported limitations included limited visualization of small perforating vessels, manual registration, absence of automatic referencing or tracking, and dependence on a stable internet connection.

Although both modalities were generated from the same underlying 3D-RA datasets, small perforating vessels were perceived as more clearly visualized in the 3D-printed models. This may have resulted from workflow-specific differences: perianeurysmal perforators were manually delineated during generation of the 3D-printed models using 3D Slicer (The Slicer Community; www.slicer.org, accessed on 3 February 2020), whereas MR-HMD visualizations were delineated using Brainlab Cranial Planning software (Brainlab AG, Munich, Germany) as described in Section 2.4.1 and Section 2.4.2, and the printed models displayed only bone and vascular structures, thereby reducing visual clutter from additional anatomical information. In contrast, MR-HMD visualization enabled dynamic three-dimensional presentation of the patient-specific anatomy (Figure 2) and the incorporation of additional MRI-derived structures if available, providing broader anatomical context but potentially reducing the visual prominence of small perforating vessels.

**Figure 4:**
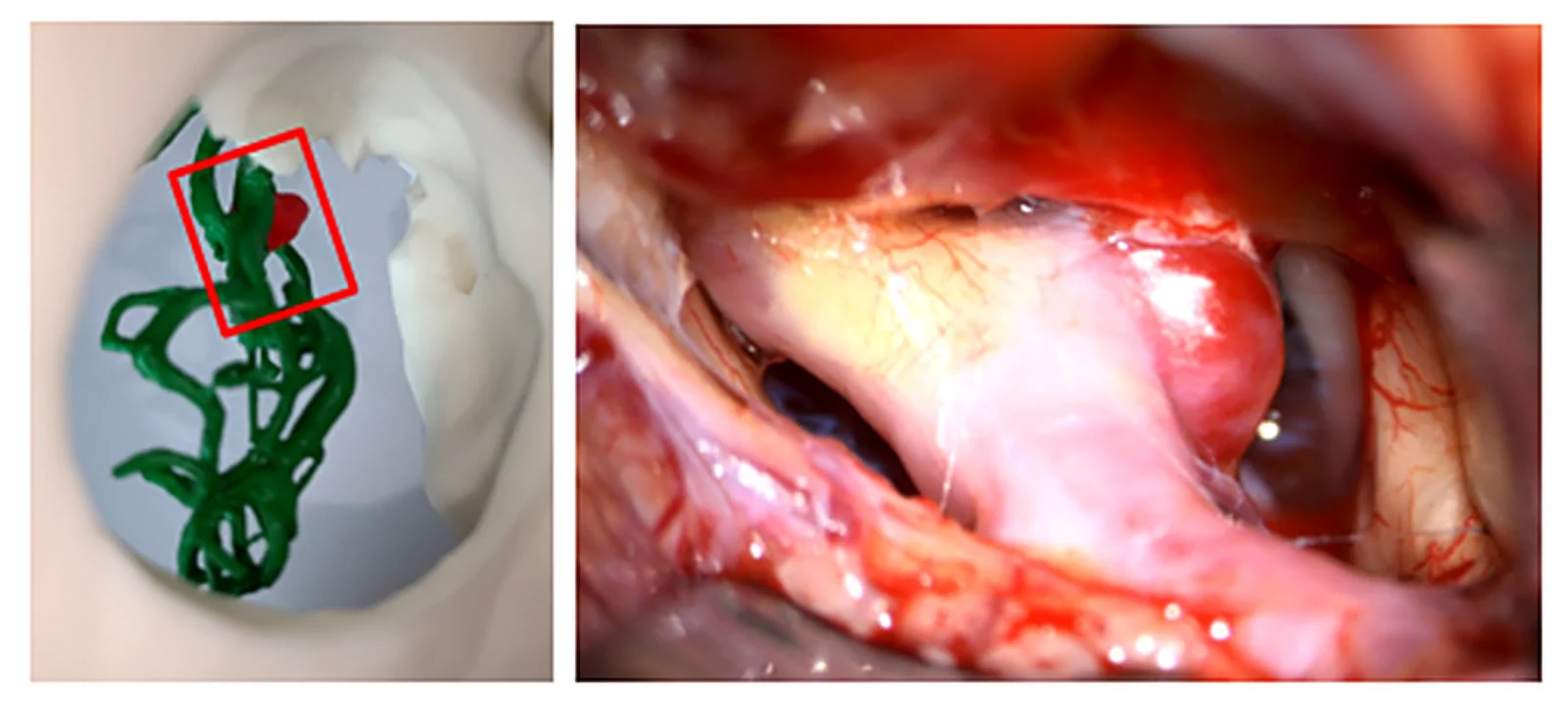
Comparison of the 3D-printed model and intraoperative microscopic view in a right-sided posterior communicating artery aneurysm. Red frame depicts the intraoperative view on the right.

Painted 3D-printed models provided additional visual differentiation of aneurysms and arteries (Figure 1). The spatial correspondence between the patient-specific 3D-printed model and the intraoperative anatomy is illustrated in Figure 4.

Because painting reduced flexibility for clip simulation, two soft hollow aneurysm models were produced for each patient: one painted model for education and one unpainted model for clip planning. A hexagonal plug-in adapter was used to position the aneurysm model inside the skull model and allowed interchange between painted and unpainted aneurysm models (Figure 1). The recurring material costs per patient were relatively low, at USD 130–150 for one skull print and two interchangeable aneurysm prints.

Several potential issues can be anticipated by future users intending to adapt our workflow. For MR-HMD visualization, the most frequent problems were the dependence on a stable internet connection in the operating room and the need to fully charge the head-mounted displays before using them. For 3D-printed models, the required isopropyl alcohol for washing the prints needs to be bought, stored, and disposed of properly; we solved this by collaborating with our hospital pharmacy. If this option is not available, one needs to check sources to buy and dispose of isopropyl alcohol according to local laws. It is also advisable to print, wash, and paint the devices in a room that is properly ventilated and to wear protective gloves while handling chemicals.

The central methodological contribution of this study is the integration of both MR-HMD visualization and 3D-printed models into a single standardized, patient-specific workflow rather than the isolated evaluation of either modality. This combined approach links routine image acquisition and fusion, modality-specific processing, and clinical integration into planning, education, and non-sterile preoperative operating room setup. By applying the same sequence of image fusion, artifact removal, modality-specific processing, and clinical integration across all patients, the workflow was designed to support reproducibility and transferability to centers with comparable neurovascular imaging infrastructure, as the additional MR-HMD and 3D-printing components are commercially available and do not depend on custom-built hardware or in-house software development.

These findings are consistent with previous reports describing the use of MR-HMD visualization and 3D-printed models in neurosurgical planning. MR-HMD-based approaches may support interactive review of patient-specific anatomy but remain limited by registration accuracy, visualization of fine structures, and lack of instrument tracking [2,7,9,11]. In contrast, 3D-printed models provide a tangible representation of patient-specific anatomy that may support clip planning, spatial orientation, simulation, and patient education, but require longer production times and are constrained by printer build volume [3,4,5,13,14]. Unlike studies focusing on either MR-HMD visualization or 3D printing alone, the present study defines and evaluates a combined workflow in which both modalities are implemented according to their respective strengths and limitations.

### 4.1. Study Limitations

This study has several limitations. First, the sample size was limited to 29 patients, and the single-center design restricted the generalizability of our findings. The present work was intentionally designed as a methodological feasibility study rather than an outcome-driven investigation; consequently, no systematic evaluation of surgical or patient-related outcomes was performed.

Second, assessment of advantages and disadvantages was based on structured evaluation forms completed by surgeons with varying levels of experience. Although both residents and attending physicians were included, the assessment reflected subjective user perception and was not based on validated social-science or human-factor instruments, usability scores or objective performance metrics.

Third, both technologies are subject to technical constraints. MR-HMD visualization currently requires manual hologram alignment and depends on stable internet connectivity for optimal rendering quality, and its effect on the total duration of preoperative operating room preparation was not measured, while 3D-printed model production is limited by printer build volume and prolonged production times.

Fourth, preprocessing software for 3D printing was updated during the study period (PreForm versions 3.3.3 to 3.10.1 and 3D Slicer version 4.10.2 with subsequent updates). The processing steps remained unchanged, but we cannot exclude that version-related differences in software performance or user interface may have influenced our recorded preparation times minimally. The fact that different software had to be used for delineation in the 3D-printed models and MR-HMD visualizations also might limit direct comparison of the two modalities. Additionally, no quantitative validation of the anatomical accuracy or dimensional fidelity of the 3D-printed models or MR-HMD visualizations against the source imaging on which they are based was performed. These limitations reflect the current state of technology and should be considered when interpreting the results.

### 4.2. Future Perspectives

Future developments should focus on reducing preprocessing effort, operator dependency, and production time and may substantially enhance the clinical utility of both MR-HMDs and 3D-printed models. Automated image segmentation and AI-assisted 3D reconstruction may reduce preprocessing time and improve reproducibility and scalability. Advances in MR-HMD technology, including improved registration algorithms, markerless tracking, and integration with optical or navigation-based instrument tracking, could enable reliable intraoperative use in the future.

Furthermore, hybrid workflows combining preoperative MR-HMD visualization with intraoperative navigation data may allow real-time updating of patient-specific models, potentially bridging the gap between planning and execution. Future analysis could include validated instruments to quantify user experience and cognitive workload (e.g., the System Usability Scale or the NASA Task Load Index, or its surgical adaptation, SURG-TLX). Larger, prospective studies incorporating clinical outcome measures will be essential to determine whether these technological advances translate into measurable improvements in surgical precision, efficiency, and patient outcomes.

## 5. Conclusions

This study presents a standardized methodological workflow for generating and clinically integrating patient-specific MR-HMD visualizations and 3D-printed models for aneurysm surgery planning. MR-HMD visualization required short preparation times and supported flexible short-term visualization during preoperative planning and non-sterile operating room setup, but was limited by incomplete visualization of small perforating vessels. In contrast, 3D-printed models required substantially longer preparation and an additional printing time of approximately 14 h 55 min, but provided a physical model for clip planning and patient education, which will be examied further in future studies analyzing quantitative measurements of both applications. These findings support the use of MR-HMD visualization and 3D-printed models as complementary components of a patient-specific planning workflow. Future work should focus on automated segmentation, improved registration, instrument tracking, larger-volume printing, and prospective evaluation of clinical and educational outcomes.

## Figures and Tables

**Table 1:** Preparation times for 3D-printed models and MR-HMD visualization.

	Minutes *mean ± SD*	*(min; max)*	(n)
**3D-printed models**			
Preprocessing	41.0 ± 9.7	(30; 60)	29
Postprocessing	67.8 ± 12.4	(45; 90)	29
Total time (excl. printing, washing, and curing)	108.8 ± 17.8	(75; 135)	29
Printing time	894.9 ± 41.6	(809; 970)	29
Washing and curing ^1^	80		
**MR-HMD**			
Preprocessing	33.5 ± 10.8	(15; 60)	29
MR-HMD setup	7.0 ± 2.8	(5; 15)	27
Total time	40.6 ± 10.3	(25; 65)	27
**Conventional navigation setup**	6.7 ± 2.1	(5; 10)	27

*^1^ Fixed protocol duration (20 min washing, 60 min curing); not measured per case.*

**Table 2:** Reported advantages and disadvantages of 3D-printed models and MR-HMD visualization.

	3D-Printed Models	MR-HMD
**Advantages**	Visibility of small perforating vessels	Visibility of other relevant structures
	Interaction (e.g., clip anticipation)	Add-on use on patient
		Adjustable visualization (zoom, rotation, add/remove structures)
**Disadvantages**	Other relevant structures besides bone and vessels not visible	Limited visibility of small perforating vessels
	Printing time	Manual registration, no automatic referencing or tracking system
	Only partial print of the skull	Dependence on internet connection

## Data Availability

The data presented in this study are available on request from the corresponding authors, in anonymized form, due to patient privacy and legal reasons.

## Supplementary Materials

The following supporting information can be downloaded at: https://www.mdpi.com/article/10.3390/brainsci16090932/s1.

## Abbreviations

The following abbreviations are used in this manuscript:

3D	Three dimensional
3D-RA	Three-dimensional rotational angiography
AI	Artificial intelligence
CT	Computed tomography
DICOM	Digital Imaging and Communications in Medicine
MR	Mixed reality
MR-HMD	Mixed-reality head-mounted display
MRI	Magnetic resonance imaging
