# Supplementary material for: Patient-Specific 3D-Printed Models and Mixed-Reality Head-Mounted Display Visualization for Aneurysm Clip Ligation Planning: A Methodological Workflow Study
Source: Brain Sci. 2026 Aug 31;16(9):932. doi: 10.3390/brainsci16090932 (PMC13604824; doi:10.3390/brainsci16090932)
Supplement: Supplementary file 1 [file brainsci-16-00932-s001.zip › brainsci-4472535-supplementary.pdf]

FORMULAR A : Patientenzufriedenheit

Patient-ID: ____________

3D-Modell Nummer: _____________

Ausbildungsstatus:

- O Keinen Schulabschluss
- O Primarschulabschluss
- O Real- oder Sekundarschulabschluss
- O Berufsschulabschluss
- O Maturitätsabschluss
- O Fachhochschulabschluss
- O Universitäts-/ETH-Abschluss
- O Anderer : ____________________________

Aktuelle Beruftätigkeit : _____________________________________

Verständnis des Patienten über den geplanten Eingriff:

Involvierte Hirnregion: ____________

Involvierte Gefässe: ____________

Mögliche Komplikationen: ____________

Geplanter Eingriff: ____________

Vergleichende Analyse der Patientenaufklärung mit konventioneller Bildgebung, Skizze vs. mit den 3D-Modellen

Bewertung: *sehr schlecht (0) – extrem gut (10)*

Mit Hilfe der konventioneller CTA Bilder

1. Wie beurteilen Sie Ihr Verständnis der Krankheit (Gefäßpathologie)?

0	1	2	3	4	5	6	7	8	9	10
sehr schlecht										*extrem gut*

2. Ich verstehe, wie mein Aneurysma aussieht

0	1	2	3	4	5	6	7	8	9	10
sehr schlecht										*extrem gut*

3. Ich verstehe, wo sich mein Aneurysma befindet

0	1	2	3	4	5	6	7	8	9	10
sehr schlecht										*extrem gut*

4. Ich verstehe mögliche Komplikationen bei der Behandlung

0	1	2	3	4	5	6	7	8	9	10
sehr schlecht										*extrem gut*

5. Ich verstehe, warum mein Chirurg die geplante Behandlung gewählt hat

0	1	2	3	4	5	6	7	8	9	10
sehr schlecht										*extrem gut*

6. Ich kann die wichtigsten Schritte zur Behandlung des Aneurysmas nennen (Kraniotomie / Aneurysmaclipping)

0	1	2	3	4	5	6	7	8	9	10
sehr schlecht										*extrem gut*

7. Ich fühle mich wohl mit dem Operationsplan

0	1	2	3	4	5	6	7	8	9	10
sehr schlecht										*extrem gut*

Mit Hilfe der konventioneller 2D-DSA Bilder

1. Wie beurteilen Sie Ihr Verständnis der Krankheit (Gefäßpathologie)?

0	1	2	3	4	5	6	7	8	9	10
sehr schlecht										*extrem gut*

2. Ich verstehe, wie mein Aneurysma aussieht

0	1	2	3	4	5	6	7	8	9	10
sehr schlecht										*extrem gut*

3. Ich verstehe, wo sich mein Aneurysma befindet

0	1	2	3	4	5	6	7	8	9	10
sehr schlecht										*extrem gut*

4. Ich verstehe mögliche Komplikationen bei der Behandlung

0	1	2	3	4	5	6	7	8	9	10
sehr schlecht										*extrem gut*

5. Ich verstehe, warum mein Chirurg die geplante Behandlung gewählt hat

0	1	2	3	4	5	6	7	8	9	10
sehr schlecht										*extrem gut*

6. Ich kann die wichtigsten Schritte zur Behandlung des Aneurysmas nennen (Kraniotomie / Aneurysmaclipping)

0	1	2	3	4	5	6	7	8	9	10
sehr schlecht										*extrem gut*

7. Ich fühle mich wohl mit dem Operationsplan

0	1	2	3	4	5	6	7	8	9	10
sehr schlecht										*extrem gut*

Mit Hilfe der 3D-DSA Bilder

1. Wie beurteilen Sie Ihr Verständnis der Krankheit (Gefäßpathologie)?

0	1	2	3	4	5	6	7	8	9	10
sehr schlecht										*extrem gut*

2. Ich verstehe, wie mein Aneurysma aussieht

0	1	2	3	4	5	6	7	8	9	10
sehr schlecht										*extrem gut*

3. Ich verstehe, wo sich mein Aneurysma befindet

0	1	2	3	4	5	6	7	8	9	10
sehr schlecht										*extrem gut*

4. Ich verstehe mögliche Komplikationen bei der Behandlung

0	1	2	3	4	5	6	7	8	9	10
sehr schlecht										*extrem gut*

5. Ich verstehe, warum mein Chirurg die geplante Behandlung gewählt hat

0	1	2	3	4	5	6	7	8	9	10
sehr schlecht										*extrem gut*

6. Ich kann die wichtigsten Schritte zur Behandlung des Aneurysmas nennen (Kraniotomie / Aneurysmaclipping)

0	1	2	3	4	5	6	7	8	9	10
sehr schlecht										*extrem gut*

7. Ich fühle mich wohl mit dem Operationsplan

0	1	2	3	4	5	6	7	8	9	10
sehr schlecht										*extrem gut*

Mit Hilfe dem 3D Virtual Reality Vector Bild:

1. Wie beurteilen Sie Ihr Verständnis der Krankheit (Gefäßpathologie)?

0	1	2	3	4	5	6	7	8	9	10
sehr schlecht										*extrem gut*

2. Ich verstehe, wie mein Aneurysma aussieht

0	1	2	3	4	5	6	7	8	9	10
sehr schlecht										*extrem gut*

3. Ich verstehe, wo sich mein Aneurysma befindet

0	1	2	3	4	5	6	7	8	9	10
sehr schlecht										*extrem gut*

4. Ich verstehe mögliche Komplikationen bei der Behandlung

0	1	2	3	4	5	6	7	8	9	10
sehr schlecht										*extrem gut*

5. Ich verstehe, warum mein Chirurg die geplante Behandlung gewählt hat

0	1	2	3	4	5	6	7	8	9	10
sehr schlecht										*extrem gut*

6. Ich kann die wichtigsten Schritte zur Behandlung des Aneurysmas nennen (Kraniotomie / Aneurysmaclipping)

0	1	2	3	4	5	6	7	8	9	10
sehr schlecht										*extrem gut*

7. Ich fühle mich wohl mit dem Operationsplan

0	1	2	3	4	5	6	7	8	9	10
sehr schlecht										*extrem gut*

<u>Mit Hilfe von dem 3D-Modell:</u>

1. Wie beurteilen Sie Ihr Verständnis der Krankheit (Gefäßpathologie)?

0	1	2	3	4	5	6	7	8	9	10
sehr schlecht										*extrem gut*

2. Ich verstehe, wie mein Aneurysma aussieht

0	1	2	3	4	5	6	7	8	9	10
sehr schlecht										*extrem gut*

3. Ich verstehe, wo sich mein Aneurysma befindet

0	1	2	3	4	5	6	7	8	9	10
sehr schlecht										*extrem gut*

4. Ich verstehe mögliche Komplikationen bei der Behandlung

0	1	2	3	4	5	6	7	8	9	10
sehr schlecht										*extrem gut*

5. Ich verstehe, warum mein Chirurg die geplante Behandlung gewählt hat

0	1	2	3	4	5	6	7	8	9	10
sehr schlecht										*extrem gut*

6. Ich kann die wichtigsten Schritte zur Behandlung des Aneurysmas nennen (Kraniotomie / Aneurysmaclipping)

0	1	2	3	4	5	6	7	8	9	10
sehr schlecht										*extrem gut*

7. Ich fühle mich wohl mit dem Operationsplan

0	1	2	3	4	5	6	7	8	9	10
sehr schlecht										*extrem gut*

<u>Mit Hilfe von der Brainlab's Magic Leap:</u>

1. Wie beurteilen Sie Ihr Verständnis der Krankheit (Gefäßpathologie)?

0	1	2	3	4	5	6	7	8	9	10
sehr schlecht										*extrem gut*

2. Ich verstehe, wie mein Aneurysma aussieht

0	1	2	3	4	5	6	7	8	9	10
sehr schlecht										*extrem gut*

3. Ich verstehe, wo sich mein Aneurysma befindet

0	1	2	3	4	5	6	7	8	9	10
sehr schlecht										*extrem gut*

4. Ich verstehe mögliche Komplikationen bei der Behandlung

0	1	2	3	4	5	6	7	8	9	10
sehr schlecht										*extrem gut*

5. Ich verstehe, warum mein Chirurg die geplante Behandlung gewählt hat

0	1	2	3	4	5	6	7	8	9	10
sehr schlecht										*extrem gut*

6. Ich kann die wichtigsten Schritte zur Behandlung des Aneurysmas nennen (Kraniotomie / Aneurysmaclipping)

0	1	2	3	4	5	6	7	8	9	10
sehr schlecht										*extrem gut*

7. Ich fühle mich wohl mit dem Operationsplan

0	1	2	3	4	5	6	7	8	9	10
sehr schlecht										*extrem gut*

Verbessertes Verständnis mit dem 3D-Modell

1. Wie hat Ihnen das 3D-Modell geholfen, etwas über Ihre Gehirnanatomie zu lernen?

0	1	2	3	4	5	6	7	8	9	10
sehr schlecht										*extrem gut*

2. Wie hat Ihnen das 3D-Modell geholfen, etwas über Ihr Aneurysma zu lernen?

0	1	2	3	4	5	6	7	8	9	10
sehr schlecht										*extrem gut*

3. Wie hat Ihnen das 3D-Modell geholfen, die Operation zu verstehen, die durchgeführt wird?

0	1	2	3	4	5	6	7	8	9	10
sehr schlecht										*extrem gut*

4. Inwiefern hat Ihnen das 3D-Modell geholfen, die möglichen Komplikationen im Zusammenhang mit dem durchzuführenden Verfahren zu verstehen

0	1	2	3	4	5	6	7	8	9	10
sehr schlecht										*extrem gut*

Fühlten Sie sich durch das Modell verunsichert? O Ja O Nein

Hat es Ängste verstärkt? O Ja O Nein

Wenn ja, durch den Schädel oder das Aneurysma?

Andere Kommentare:

Verbessertes Verständnis mit Brainlab Magic Leap:

1. Wie hat Ihnen das 3D-Modell geholfen, etwas über Ihre Gehirnanatomie zu lernen?

0	1	2	3	4	5	6	7	8	9	10
sehr schlecht										*extrem gut*

2. Wie hat Ihnen das 3D-Modell geholfen, etwas über Ihr Aneurysma zu lernen?

0	1	2	3	4	5	6	7	8	9	10
sehr schlecht										*extrem gut*

3. Wie hat Ihnen das 3D-Modell geholfen, die Operation zu verstehen, die durchgeführt wird?

0	1	2	3	4	5	6	7	8	9	10
sehr schlecht										*extrem gut*

4. Inwiefern hat Ihnen das 3D-Modell geholfen, die möglichen Komplikationen im Zusammenhang mit dem durchzuführenden Verfahren zu verstehen

0	1	2	3	4	5	6	7	8	9	10
sehr schlecht										*extrem gut*

Fühlten Sie sich durch das Modell verunsichert? O Ja O Nein

Hat es Ängste verstärkt? O Ja O Nein

Wenn ja, durch den Schädel oder das Aneurysma?

Andere Kommentare:

Wie evaluieren Sie die Technik auf einer Skala von 0 bis 10 (1= sehr schlecht und 10=excellent)

CTA:

0	1	2	3	4	5	6	7	8	9	10
sehr schlecht										*extrem gut*

DSA 2D:

0	1	2	3	4	5	6	7	8	9	10
sehr schlecht										*extrem gut*

DSA 3D:

0	1	2	3	4	5	6	7	8	9	10
sehr schlecht										*extrem gut*

Vector Bild (3D)

0	1	2	3	4	5	6	7	8	9	10
sehr schlecht										*extrem gut*

3D printed Modell

0	1	2	3	4	5	6	7	8	9	10
sehr schlecht										*extrem gut*

Magic Leap

0	1	2	3	4	5	6	7	8	9	10
sehr schlecht										*extrem gut*

Wenn Sie nur 1 Technik zur Verfügung hätten, welche hätten Sie lieber gehabt:

O CTA

O DSA

O 3D-Vector

O 3D-Modell

O Brainlab’s Magic Leap

FORMULAR B : Clip Anticipation

Patient-ID: ____________

3D-Modell Number: _____________

Examiner-ID (senior, junior, resident): ____________

Name of resident: ____________

Preoperative assessment

IA reconstruction based on preoperative radiological imaging:

Single clip (y/n): __________ Multiple clips (y/n) :_____________

Serial clips (y/n): __________

Choice of clip (nr.): ___________

Perforator at risk for temporary clipping (y/n): ___________

Additional wrapping needed (y/n): ____________

IA reconstruction based on printed 3D-model:

Single clip (y/n): __________ Multiple clips (y/n) :_____________

Serial clips (y/n): __________

Choice of clip (nr.): __________

Perforator at risk for temporary clipping (y/n): _____________

Additional wrapping needed (y/n): _______________

IA reconstruction based on Brainlab Magic Leap:

Single clip (y/n): __________ Multiple clips (y/n) :_____________

Serial clips (y/n): __________

Choice of clip (nr.): __________

Perforator at risk for temporary clipping (y/n): _____________

Additional wrapping needed (y/n): _______________

Number of attempts: ___________

Attempt 1: _____________

Remnant (Sindou I-V): _________

Single clip (y/n): __________ Multiple clips (y/n) :_____________

Serial clips (y/n): __________

Choice of clip(s) (nr.): _______________________________________

➔ Take a photograph

Attempt 2: _____________

Remnant (Sindou I-V): _________

Single clip (y/n): __________ Multiple clips (y/n) :_____________

Serial clips (y/n): __________

Choice of clip(s) (nr.): _______________________________________

➔ Take a photograph

Attempt 3: _____________

Remnant (Sindou I-V): _________

Single clip (y/n): __________ Multiple clips (y/n) :_____________

Serial clips (y/n): __________

Choice of clip(s) (nr.): _______________________________________

➔ Take a photograph

>3 attempts: ____________

➔ Take a photograph

Intraoperative assessment

Temporary clipping time (min:sec): ____

Repositioning after visual inspection (y/n): _________

➔ Take a photograph

Repositioning after Doppler (y/n): _________

Repositioning after ICG (y/n): _________

➔ Take a photograph

Repositioning after 3D-iDSA (y/n): _________

➔ Take a photograph

Additional wrapping needed (y/n): _________

Single clip (y/n): __________ Multiple clips (y/n) :_____________

Serial clips (y/n): __________

Choice of clip(s) (nr.): ______________________________________

Perforator at risk for temporary clipping (y/n): _____________

IA remnant in 3D-iDSA (Sindou I-V): __________

Postoperative assessment

Ischemia in 24h postop CT: _____________

Neurological deficit: _________________

Other complication: __________________

Abbreviations

IA = intracranial aneurysm; MCA = middle cerebral artery; AComA = anterior communicating artery; ICA = internal carotid artery; ACA = proximal anterior cerebral artery; PComA = posterior communicating artery; ACha = anterior choroidal artery; BA = basilar artery.

Sindou Classification

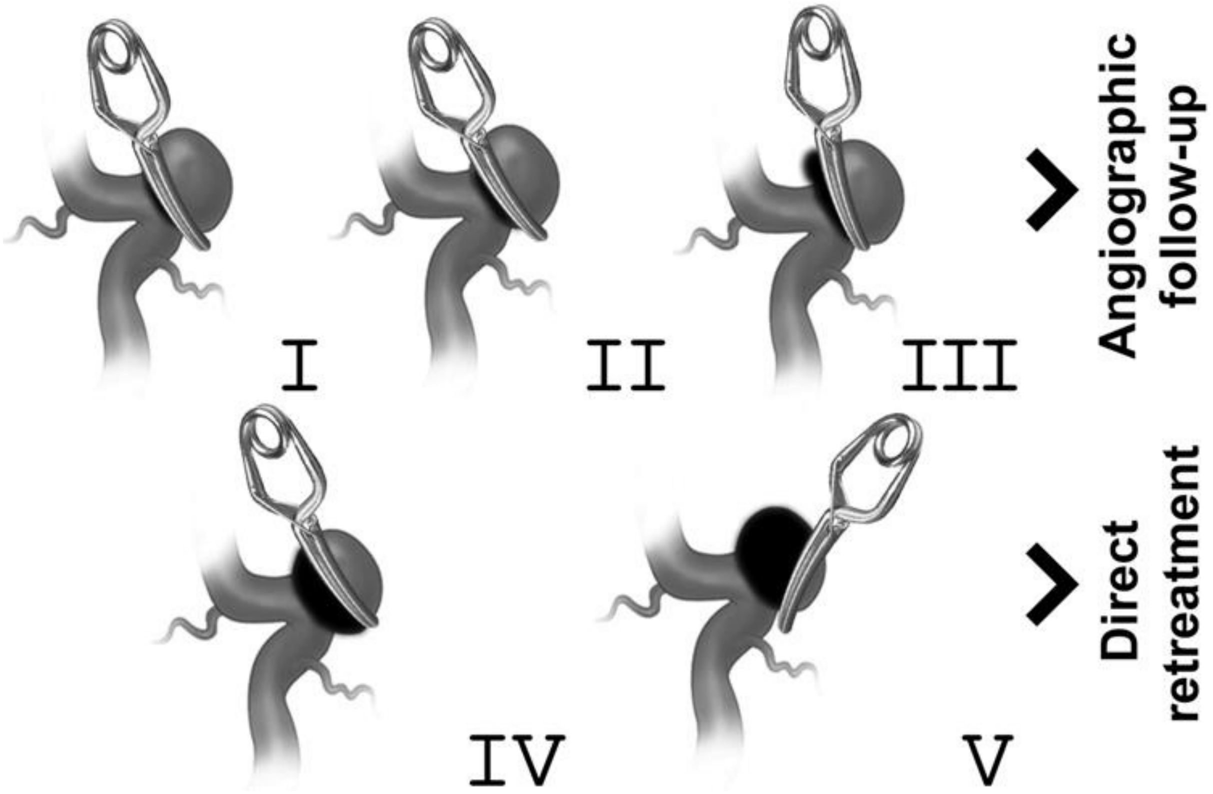

Grade I: remnant less than 50% of the neck implantation
Grade II: remnant more than 50% of the neck implantation
Grade III: residual lobe from a multilobulated sac
Grade IV: residual portion of the sac, less than 75% of the aneurysmal size
Grade V: residual portion of the sac more than 75% of the aneurysmal size.

FORMULAR C : Residents Teaching

Patient-ID: ___________________ 3D-Modell Nummer: ______________

Junior Resident's year in neurosurgery: ___________________________

PART 1: Understanding of the disease

Based on CTA/MRA:

1) Aneurysm location:

ICA:
- o C1 – cervical
- o C2 – petrous
- o C3 – lacerum
- o C4 – cavernous
- o C5 – clinoidal
- o C6 – ophthalmic
- o C7 – communicating
- o Anterior choroidal anterior

ACA:
- o A1 – precommunicating (horizontal)
- o A2 – postcommunicting (infracallosal)
- o A3 – precallosal
- o A4 – supracallosal
- o A5 – postcallosal

MCA:
- o M1 – sphenoidal
- o M2 – insular
- o M3 – opercular
- o M4 – cortical

VA:
- o V1 – pre-foraminal
- o V2 – foraminla
- o V3 – extradural
- o V4 – intradural

SCA:
- o s1 – anterior pontomesencephalic
- o s2 – lateral pontomesencephalic
- o s3 – cerebellomesencephalic
- o s4 – cortical

AICA:
- o a1- anterior pontine
- o a2 – lateral pontomesencephalic
- o a3 – flocculopeduncular
- o a4 – cortical

PICA:
- o p1 – anterior medullary
- o p2 – lateral medullary
- o p3 – tonsillomedullary
- o p4 – telovelotonsillar
- o p5 – cortical

BA:
- o Basilar tip
- o other

PCA :
- o P1 – precommunicating
- o P2 – postcommunicating
- o P2A – crural
- o P2P – ambient
- o P3 – quadrigeminal
- o P4 – calcarine

2) Shape:
- o Fusiform
- o Saccular
- o Multilobulated
- o Broad-based

3) Relevant adhering vessels
- o Yes – arterial branch originating in the aneurysm pouch
- o Yes – arterial branch from elsewhere
- o Yes – venous structure
- o unclear
- o No

4) Perforators at risk

- ○ Yes, with definitive clip
- ○ Yes, with temporary clip
- ○ No

5) Relevant Surrounding Structures

<u>Nerves:</u>

- ○ CN I
- ○ CN II
- ○ CN III
- ○ CN IV
- ○ CN V
- ○ VI
- ○ CN VII
- ○ CN VIII
- ○ CN IX
- ○ CN X
- ○ CN XI
- ○ CN XII
- ○ GSPN
- ○ No

<u>Bony Structures:</u>

- ○ Greater Sphenoid wing
- ○ Lesser Sphenoid wing
- ○ Sphenoid plane
- ○ other
- ○ No

6) Presence of Calcifications

- ○ Yes, intraaneurysmal
- ○ Yes, at clip site
- ○ Yes, at parent vessel
- ○ No

7) Anterior Clinoidectomy needed:

- ○ Yes
- ○ No

8) Temporary Clip position:

- ○ A1 links
- ○ A1 rechts
- ○ M1 links
- ○ M1 rechts
- ○ ICA links
- ○ ICA rechts

Based on DSA:

1) Aneurysm location:

ICA:
- o C1 – cervical
- o C2 – petrous
- o C3 – lacerum
- o C4 – cavernous
- o C5 – clinoidal
- o C6 – ophthalmic
- o C7 – communicating
- o Anterior choroidal anterior

ACA:
- o A1 – precommunicating (horizontal)
- o A2 – postcommunicting (infracallosal)
- o A3 – precallosal
- o A4 – supracallosal
- o A5 – postcallosal

MCA:
- o M1 – sphenoidal
- o M2 – insular
- o M3 – opercular
- o M4 – cortical

VA:
- o V1 – pre-foraminal
- o V2 – foraminla
- o V3 – extradural
- o V4 – intradural

SCA:
- o s1 – anterior pontomesencephalic
- o s2 – lateral pontomesencephalic
- o s3 – cerebellomesencephalic
- o s4 – cortical

AICA:
- o a1- anterior pontine
- o a2 – lateral pontomesencephalic
- o a3 – flocculopeduncular
- o a4 – cortical

PICA:
- o p1 – anterior medullary
- o p2 – lateral medullary
- o p3 – tonsillomedullary
- o p4 – telovelotonsillar
- o p5 – cortical

BA:
- o Basilar tip
- o other

PCA :
- o P1 – precommunicating
- o P2 – postcommunicating
- o P2A – crural
- o P2P – ambient
- o P3 – quadrigeminal
- o P4 – calcarine

2) Shape:
- o Fusiform
- o Saccular
- o Multilobulated
- o Broad-based

3) Relevant adhering vessels
- o Yes – arterial branch originating in the aneurysm pouch
- o Yes – arterial branch from elsewhere
- o Yes – venous structure
- o unclear
- o No

4) Perforators at risk
- o Yes, with definitive clip
- o Yes, with temporary clip
- o No

5) Relevant Surrounding Structures

Nerves:

- CN I
- CN II
- CN III
- CN IV
- CN V
- VI
- CN VII
- CN VIII
- CN IX
- CN X
- CN XI
- CN XII
- GSPN
- No

Bony Structures:

- Greater Sphenoid wing
- Lesser Sphenoid wing
- Sphenoid plane
- other
- No

6) Presence of Calcifications

- Yes, intraaneurysmal
- Yes, at clip site
- Yes, at parent vessel
- No

7) Anterior Clinoidectomy needed:

- Yes
- No

8) Temporary Clip position:

- A1 links
- A1 rechts
- M1 links
- M1 rechts
- ICA links
- ICA rechts

Based on 3D vector picture:

1) Aneurysm location:

ICA:
- C1 – cervical
- C2 – petrous
- C3 – lacerum
- C4 – cavernous
- C5 – clinoidal
- C6 – ophthalmic
- C7 – communicating
- Anterior choroidal anterior

ACA:
- A1 – precommunicating (horizontal)
- A2 – postcommunicting (infracallosal)
- A3 – precallosal
- A4 – supracallosal
- A5 – postcallosal

MCA:
- M1 – sphenoidal
- M2 – insular
- M3 – opercular
- M4 – cortical

VA:
- V1 – pre-foraminal
- V2 – foraminla
- V3 – extradural
- V4 – intradural

SCA:
- s1 – anterior pontomesencephalic
- s2 – lateral pontomesencephalic
- s3 – cerebellomesencephalic
- s4 – cortical

AICA:
- a1- anterior pontine
- a2 – lateral pontomesencephalic
- a3 – flocculopeduncular
- a4 – cortical

PICA:
- p1 – anterior medullary
- p2 – lateral medullary
- p3 – tonsillomedullary
- p4 – telovelotonsillar
- p5 – cortical

BA:
- Basilar tip
- other

PCA :
- P1 – precommunicating
- P2 – postcommunicating
- P2A – crural
- P2P – ambient
- P3 – quadrigeminal
- P4 – calcarine

2) Shape:
- Fusiform
- Saccular
- Multilobulated
- Broad-based

3) Relevant adhering vessels
- Yes – arterial branch originating in the aneurysm pouch
- Yes – arterial branch from elsewhere
- Yes – venous structure
- unclear
- No

4) Perforators at risk
- Yes, with definitive clip
- Yes, with temporary clip
- No

5) Relevant Surrounding Structures

Nerves:

- o CN I
- o CN II
- o CN III
- o CN IV
- o CN V
- o VI
- o CN VII
- o CN VIII
- o CN IX
- o CN X
- o CN XI
- o CN XII
- o GSPN
- o No

Bony Structures:

- o Greater Sphenoid wing
- o Lesser Sphenoid wing
- o Sphenoid plane
- o other
- o No

6) Presence of Calcifications

- o Yes, intraaneurysmal
- o Yes, at clip site
- o Yes, at parent vessel
- o No

7) Anterior Clinoidectomy needed:

- o Yes
- o No

8) Temporary Clip position:

- o A1 links
- o A1 rechts
- o M1 links
- o M1 rechts
- o ICA links
- o ICA rechts

Based on 3D printed model:

9) Aneurysm location:

ICA:
- C1 – cervical
- C2 – petrous
- C3 – lacerum
- C4 – cavernous
- C5 – clinoidal
- C6 – ophthalmic
- C7 – communicating
- Anterior choroidal anterior

ACA:
- A1 – precommunicating (horizontal)
- A2 – postcommunicting (infracallosal)
- A3 – precallosal
- A4 – supracallosal
- A5 – postcallosal

MCA:
- M1 – sphenoidal
- M2 – insular
- M3 – opercular
- M4 – cortical

VA:
- V1 – pre-foraminal
- V2 – foraminla
- V3 – extradural
- V4 – intradural

SCA:
- s1 – anterior pontomesencephalic
- s2 – lateral pontomesencephalic
- s3 – cerebellomesencephalic
- s4 – cortical

AICA:
- a1- anterior pontine
- a2 – lateral pontomesencephalic
- a3 – flocculopeduncular
- a4 – cortical

PICA:
- p1 – anterior medullary
- p2 – lateral medullary
- p3 – tonsillomedullary
- p4 – telovelotonsillar
- p5 – cortical

BA:
- Basilar tip
- other

PCA :
- P1 – precommunicating
- P2 – postcommunicating
- P2A – crural
- P2P – ambient
- P3 – quadrigeminal
- P4 – calcarine

10) Shape:
- Fusiform
- Saccular
- Multilobulated
- Broad-based

11) Relevant adhering vessels
- Yes – arterial branch originating in the aneurysm pouch
- Yes – arterial branch from elsewhere
- Yes – venous structure
- unclear
- No

12) Perforators at risk
- Yes, with definitive clip
- Yes, with temporary clip
- No

13) Relevant Surrounding Structures

Nerves:

- CN I
- CN II
- CN III
- CN IV
- CN V
- VI
- CN VII
- CN VIII
- CN IX
- CN X
- CN XI
- CN XII
- GSPN
- No

Bony Structures:

- Greater Sphenoid wing
- Lesser Sphenoid wing
- Sphenoid plane
- other
- No

14) Presence of Calcifications

- Yes, intraaneurysmal
- Yes, at clip site
- Yes, at parent vessel
- No

15) Anterior Clinoidectomy needed:

- Yes
- No

16) Temporary Clip position:

- A1 links
- A1 rechts
- M1 links
- M1 rechts
- ICA links
- ICA rechts

Based on Brainlab Magic Leap :

1) Aneurysm location:

ICA:
- C1 – cervical
- C2 – petrous
- C3 – lacerum
- C4 – cavernous
- C5 – clinoidal
- C6 – ophthalmic
- C7 – communicating
- Anterior choroidal anterior

ACA:
- A1 – precommunicating (horizontal)
- A2 – postcommunicting (infracallosal)
- A3 – precallosal
- A4 – supracallosal
- A5 – postcallosal

MCA:
- M1 – sphenoidal
- M2 – insular
- M3 – opercular
- M4 – cortical

VA:
- V1 – pre-foraminal
- V2 – foraminla
- V3 – extradural
- V4 – intradural

SCA:
- s1 – anterior pontomesencephalic
- s2 – lateral pontomesencephalic
- s3 – cerebellomesencephalic
- s4 – cortical

AICA:
- a1- anterior pontine
- a2 – lateral pontomesencephalic
- a3 – flocculopeduncular
- a4 – cortical

PICA:
- p1 – anterior medullary
- p2 – lateral medullary
- p3 – tonsillomedullary
- p4 – telovelotonsillar
- p5 – cortical

BA:
- Basilar tip
- other

PCA :
- P1 – precommunicating
- P2 – postcommunicating
- P2A – crural
- P2P – ambient
- P3 – quadrigeminal
- P4 – calcarine

2) Shape:
- Fusiform
- Saccular
- Multilobulated
- Broad-based

3) Relevant adhering vessels
- Yes – arterial branch originating in the aneurysm pouch
- Yes – arterial branch from elsewhere
- Yes – venous structure
- unclear
- No

4) Perforators at risk
- Yes, with definitive clip
- Yes, with temporary clip
- No

5) Relevant Surrounding Structures

Nerves:

- CN I
- CN II
- CN III
- CN IV
- CN V
- VI
- CN VII
- CN VIII
- CN IX
- CN X
- CN XI
- CN XII
- GSPN
- No

Bony Structures:

- Greater Sphenoid wing
- Lesser Sphenoid wing
- Sphenoid plane
- other
- No

6) Presence of Calcifications

- Yes, intraaneurysmal
- Yes, at clip site
- Yes, at parent vessel
- No

7) Anterior Clinoidectomy needed:

- Yes
- No

8) Temporary Clip position:

- A1 links
- A1 rechts
- M1 links
- M1 rechts
- ICA links
- ICA rechts

Intraoperative findings:

1) Aneurysm location:

ICA:

- C1 – cervical
- C2 – petrous
- C3 – lacerum
- C4 – cavernous
- C5 – clinoidal
- C6 – ophthalmic
- C7 – communicating
- Anterior choroidal anterior

ACA:

- A1 – precommunicating (horizontal)
- A2 – postcommunicting (infracallosal)
- A3 – precallosal
- A4 – supracallosal
- A5 – postcallosal

MCA:

- M1 – sphenoidal
- M2 – insular
- M3 – opercular
- M4 – cortical

VA:

- V1 – pre-foraminal
- V2 – foraminla
- V3 – extradural
- V4 – intradural

SCA:

- s1 – anterior pontomesencephalic
- s2 – lateral pontomesencephalic
- s3 – cerebellomesencephalic
- s4 – cortical

AICA:

- a1- anterior pontine
- a2 – lateral pontomesencephalic
- a3 – flocculopeduncular
- a4 – cortical

PICA:

- p1 – anterior medullary
- p2 – lateral medullary
- p3 – tonsillomedullary
- p4 – telovelotonsillar
- p5 – cortical

BA:

- Basilar tip
- other

PCA :

- P1 – precommunicating
- P2 – postcommunicating
- P2A – crural
- P2P – ambient
- P3 – quadrigeminal
- P4 – calcarine

2) Shape:

- Fusiform
- Saccular
- Multilobulated
- Broad-based

3) Relevant adhering vessels

- Yes – arterial branch originating in the aneurysm pouch
- Yes – arterial branch from elsewhere
- Yes – venous structure
- unclear
- No

4) Perforators at risk

- Yes, with definitive clip
- Yes, with temporary clip
- No

5) Relevant Surrounding Structures

Nerves:

- CN I
- CN II
- CN III
- CN IV
- CN V
- VI
- CN VII
- CN VIII
- CN IX
- CN X
- CN XI
- CN XII
- GSPN
- No

Bony Structures:

- Greater Sphenoid wing
- Lesser Sphenoid wing
- Sphenoid plane
- other
- No

6) Presence of Calcifications

- Yes, intraaneurysmal
- Yes, at clip site
- Yes, at parent vessel
- No

7) Anterior Clinoidectomy needed:

- Yes
- No

8) Temporary Clip position:

- A1 links
- A1 rechts
- M1 links
- M1 rechts
- ICA links
- ICA rechts

PART 2: Self-assessment: How the technique helps me to understand

0 = very bad

10 = very good

CTA/MRA:

Aneurysm Localization / parent Vessel:

0	1	2	3	4	5	6	7	8	9	10
sehr schlecht										*extrem gut*

Aneurysm Shape:

0	1	2	3	4	5	6	7	8	9	10
sehr schlecht										*extrem gut*

Relevant adhering vessels:

0	1	2	3	4	5	6	7	8	9	10
sehr schlecht										*extrem gut*

Perforators at risk:

0	1	2	3	4	5	6	7	8	9	10
sehr schlecht										*extrem gut*

Relevant surrounding structures:

0	1	2	3	4	5	6	7	8	9	10
sehr schlecht										*extrem gut*

DSA:

Aneurysm Localization / parent Vessel:

0	1	2	3	4	5	6	7	8	9	10
sehr schlecht										*extrem gut*

Aneurysm Shape:

0	1	2	3	4	5	6	7	8	9	10
sehr schlecht										*extrem gut*

Relevant adhering vessels:

0	1	2	3	4	5	6	7	8	9	10
sehr schlecht										*extrem gut*

Perforators at risk:

0	1	2	3	4	5	6	7	8	9	10
sehr schlecht										*extrem gut*

Relevant surrounding structures:

0	1	2	3	4	5	6	7	8	9	10
sehr schlecht										*extrem gut*

3D-Vector Picture:

Aneurysm Localization / parent Vessel:

0	1	2	3	4	5	6	7	8	9	10
sehr schlecht										*extrem gut*

Aneurysm Shape:

0	1	2	3	4	5	6	7	8	9	10
sehr schlecht										*extrem gut*

Relevant adhering vessels:

0	1	2	3	4	5	6	7	8	9	10
sehr schlecht										*extrem gut*

Perforators at risk:

0	1	2	3	4	5	6	7	8	9	10
sehr schlecht										*extrem gut*

Relevant surrounding structures:

0	1	2	3	4	5	6	7	8	9	10
sehr schlecht										*extrem gut*

3D-Modell:

Aneurysm Localization / parent Vessel:

0	1	2	3	4	5	6	7	8	9	10
sehr schlecht										*extrem gut*

Aneurysm Shape:

0	1	2	3	4	5	6	7	8	9	10
sehr schlecht										*extrem gut*

Relevant adhering vessels:

0	1	2	3	4	5	6	7	8	9	10
sehr schlecht										*extrem gut*

Perforators at risk:

0	1	2	3	4	5	6	7	8	9	10
sehr schlecht										*extrem gut*

Relevant surrounding structures:

0	1	2	3	4	5	6	7	8	9	10
sehr schlecht										*extrem gut*

Brainlab Magic Leap:

Aneurysm Localization / parent Vessel:

0	1	2	3	4	5	6	7	8	9	10
sehr schlecht										*extrem gut*

Aneurysm Shape:

0	1	2	3	4	5	6	7	8	9	10
sehr schlecht										*extrem gut*

Relevant adhering vessels:

0	1	2	3	4	5	6	7	8	9	10
sehr schlecht										*extrem gut*

Perforators at risk:

0	1	2	3	4	5	6	7	8	9	10
sehr schlecht										*extrem gut*

Relevant surrounding structures:

0	1	2	3	4	5	6	7	8	9	10
sehr schlecht										*extrem gut*

If you have to choose only ONE tool in order to plan and prepare you for the OP you will choose:

O CTA

O DSA

O 3D-Vector

O 3D-Modell

O Brainlb’s Magic Leap

PART 3: Planning clip choice

Junior Resident’s year in neurosurgery: ___________________________

Clip choice: how many Clip/s and Clip Reference Number

Anticipated by junior resident: ____________________________________
Picture documentation:

Anticipated by senior resident: ___________________________________
Picture documentation:

Anticipated by attending surgeon:________________________________
Picture documentation:

Anticipated by consultant (clipper):________________________________
Picture documentation:

Effectively used clip: ____
Picture documentation:

FORMULAR B : Clip Anticipation

Patient-ID: ____________

3D-Modell Number: _____________

Examiner-ID (senior, junior, resident): ____________

Name of resident: ____________

Preoperative assessment

IA reconstruction based on preoperative radiological imaging:

Single clip (y/n): __________ Multiple clips (y/n) :_____________

Serial clips (y/n): __________

Choice of clip (nr.): ___________

Perforator at risk for temporary clipping (y/n): ___________

Additional wrapping needed (y/n): ____________

IA reconstruction based on printed 3D-model:

Single clip (y/n): __________ Multiple clips (y/n) :_____________

Serial clips (y/n): __________

Choice of clip (nr.): __________

Perforator at risk for temporary clipping (y/n): _____________

Additional wrapping needed (y/n): _______________

IA reconstruction based on Brainlab Magic Leap:

Single clip (y/n): ___________ Multiple clips (y/n) :______________

Serial clips (y/n): ___________

Choice of clip (nr.): ___________

Perforator at risk for temporary clipping (y/n): ______________

Additional wrapping needed (y/n): ________________

Number of attempts: ___________

Attempt 1: _____________

Remnant (Sindou I-V): _________

Single clip (y/n): __________ Multiple clips (y/n) :_____________

Serial clips (y/n): __________

Choice of clip(s) (nr.): _______________________________________

➔ Take a photograph

Attempt 2: _____________

Remnant (Sindou I-V): _________

Single clip (y/n): __________ Multiple clips (y/n) :_____________

Serial clips (y/n): __________

Choice of clip(s) (nr.): _______________________________________

➔ Take a photograph

Attempt 3: _____________

Remnant (Sindou I-V): _________

Single clip (y/n): __________ Multiple clips (y/n) :_____________

Serial clips (y/n): __________

Choice of clip(s) (nr.): _______________________________________

➔ Take a photograph

>3 attempts: ____________

➔ Take a photograph

Intraoperative assessment

Temporary clipping time (min:sec): ____

Repositioning after visual inspection (y/n): _________

- ➔ Take a photograph

Repositioning after Doppler (y/n): _________

Repositioning after ICG (y/n): _________

- ➔ Take a photograph

Repositioning after 3D-iDSA (y/n): _________

- ➔ Take a photograph

Additional wrapping needed (y/n): _________

Single clip (y/n): __________ Multiple clips (y/n) :_____________

Serial clips (y/n): __________

Choice of clip(s) (nr.): ______________________________________

Perforator at risk for temporary clipping (y/n): _____________

IA remnant in 3D-iDSA (Sindou I-V): __________

Postoperative assessment

Ischemia in 24h postop CT: _____________

Neurological deficit: _________________

Other complication: __________________

Abbreviations

IA = intracranial aneurysm; MCA = middle cerebral artery; AComA = anterior communicating artery; ICA = internal carotid artery; ACA = proximal anterior cerebral artery; PComA = posterior communicating artery; ACha = anterior choroidal artery; BA = basilar artery.

Sindou Classification

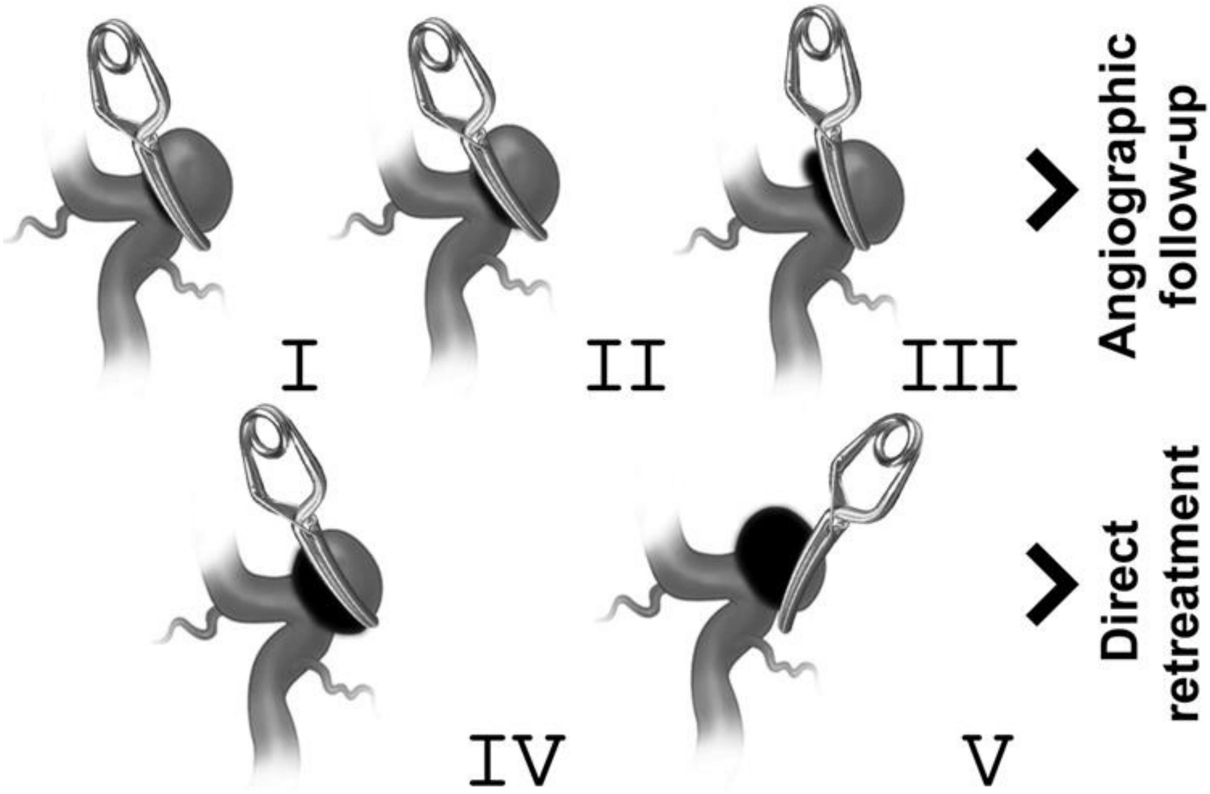

Grade I: remnant less than 50% of the neck implantation
Grade II: remnant more than 50% of the neck implantation
Grade III: residual lobe from a multilobulated sac
Grade IV: residual portion of the sac, less than 75% of the aneurysmal size
Grade V: residual portion of the sac more than 75% of the aneurysmal size.
